# Frame Analysis: An Inclusive Stakeholder Analysis Tool for Companion Animal Management in Remote Aboriginal Communities

**DOI:** 10.3390/ani11030613

**Published:** 2021-02-26

**Authors:** Brooke P. A. Kennedy, Wendy Y. Brown, James R. A. Butler

**Affiliations:** 1 School of Environmental and Rural Science, University of New England, Armidale, NSW 2353, Australia; wbrown@une.edu.au; 2 CSIRO Land & Water, GPO Box 2583, Brisbane, QLD 4001, Australia; james.butler@csiro.au

**Keywords:** animal management, collective learning, dog overpopulation, Indigenous resource management, knowledge cultures, power frames, stakeholder participation

## Abstract

**Simple Summary:**

The management of dogs and cats in remote communities is challenging due to limited availability of veterinary services and high reproductive rates in companion animal populations. Support for animal management within communities is also weak, and consequently, programs delivered by external providers rarely achieve sustainable outcomes. An alternative approach whereby communities are engaged in analysing and understanding the issues, and designing solutions themselves, may help to achieve sustainable improvements in animal populations and health management. To test this approach, stakeholders involved with animal management in a remote Australian Aboriginal community were interviewed to gain their perspectives on animal management. By applying frame analysis to understand stakeholders’ perceptions, knowledge and power, interviewees fell into four distinct groups: Indigenous Locals, Indigenous Rangers, Non-Indigenous Locals and Animal Managers. The most important issue identified by all groups was the overpopulation of dogs, but there were differences in their framing of the problem and its causes. Frame analysis achieved the important first step of the process, identifying “What is the issue?”.

**Abstract:**

Companion animal management in Australian remote Aboriginal communities (rAcs) is a complex problem, with multiple stakeholders involved with differing needs, knowledge, power and resources. We present our CoMM4Unity approach, a participatory systemic action research process designed to address such problems. In the first step, frame analysis is used to analyse stakeholders’ perspectives, knowledge types and power dynamics to determine their relative roles in animal management. Twenty individuals were interviewed from stakeholder groups involved in animal management in the remote, island rAc of Wurrumiyanga, Tiwi Islands. Frame analysis indicated that stakeholders aligned into four groups with distinct identity frames, knowledge types and power frames: Indigenous Locals, Indigenous Rangers, Non-Indigenous Locals and Animal Managers. All four groups shared overlapping perceptions about companion animals in Wurrumiyanga, and agreed that dog overpopulation was the primary issue. However, the groups differed in their strength of opinions about how dogs should be managed. Therefore, the situation is not one of diametrically opposing frames but more a misalignment of goals and values. Our application showed that frame analysis can reveal subtle variations in stakeholder groups’ identities, goals and values, and hence how they prioritise management measures.

## 1. Introduction

Conflict between self-interest and the interest of the collective has a long history [1]. These competing objectives are the basis of environmental disputes and natural resource conflicts [2,3], and despite exhaustive efforts, many remain unresolved. Stakeholders whose needs are not met, usually through lack of involvement during the decision-making process, lose interest in the organisations which have failed to recognise the multiple interests in the resource [4]. Differences amongst stakeholders’ values and morals also contribute to the intractability of such conflicts [3]. Many environmental conflicts can therefore be termed “wicked problems”, which have multiple understandings from different stakeholders with differing needs, meaning that there is no true resolution and therefore increasing conflict over a persistent and intractable issue, often causing further, multi-faceted problems [5]. This is exacerbated by stakeholders tending to tackle the manageable components, or symptoms, but ignore the root causes of the problem [6].

The evolution of a natural resource conflict may be reversed via stakeholder participation. Reed [7] identified eight features of best practice for participation for this process to be successful, with the fundamental objective of embracing the diversity of knowledge and values of all individual stakeholders by involving them in the entire process. Brown and Lambert [8] suggest that such individual thinking should be extended via collective social learning so that stakeholders can tackle more complex problems because multiple solutions designed from different perspectives are necessary. Over time [8,9,10], knowledge cultures emerge based on individuals’ experiences: individual, local, specialised and organisational, and if combined, they create holistic knowledge, whereby a unifying shared focus can provide direction under conditions of complexity, uncertainty and risk (Table 1). However, different knowledge cultures have varied primacy and power, which must be understood and managed if collective learning is to take place [11]. Importantly, the specific relationships between knowledge cultures and power will vary from context to context [12].

Conflicting power frames or hierarchal conflicts enhance the division and longevity of intractable conflicts [13], since access to knowledge can be significantly affected by power [14]. This is particularly evident when working in contexts involving traditional or Indigenous knowledge relating to natural resources, not least in Australia [15,16]. As a result, alternative approaches to research have been developed that work “with” Indigenous communities to co-manage research collectively [17], forming cross-cultural studies that can embrace similarities and differences between knowledge cultures, and integrate them to generate more holistic knowledge [18,19,20].

Originally coined by Bateson [21] in 1955, frame analysis has been widely applied across many disciplines including public policy analysis, artificial intelligence, psychology, linguistics, social movement studies, communication studies, dispute resolution, music [22] and health [23,24]. Framing is the process of constructing our interpretations of the world around us by categorising our experiences, particularly against our own previous interpretations [25]. Frame analysis is used to understand the perspectives, knowledge, values and interests of all stakeholders to define the issue, determine what needs to be accomplished, justify a stance on the issue and mobilise people to take action [25].

A modified method for frame analysis applied to environmental disputes was published by Gray [25], which emphasised a need for a more systematic analysis using multiple frames. Of these, power frames, identity frames, whole-story frames and characterisation frames are four cognitive frames that are deemed highly influential in environmental disputes (Table 2), because they are salient, critical and strong determinants of the dynamics of conflicts [25,26]. Within them, Gray [25] identified nine categories for power frames (Table 2) and five categories for identity frames, which relate to self-identity. Challenges of identity question the beliefs and values of an individual, threatening their identity and almost inevitably causing conflict [25]. Whole-story frames provide a summary of what each group believes the conflict is about. Characterisation frames ask the question, “who are the other parties”? In a conflict situation, these are generally negative views about opposing groups arising from the attribution of blame and causality that are based on experiences of the other parties [25].

In remote Aboriginal communities (rAcs) of northern Australia, the issue of domestic dog management is complex and historically contentious. In this situation, multi-stakeholder participation, knowledge cultures and Indigenous resource management are equally relevant. High and growing numbers of dogs and cats in rAcs live in close proximity to humans [27,28,29], generating animal health problems, risks to human health [30,31,32,33], wildlife [34,35,36,37], and a potential reservoir for rabies should the disease become established in Australia [38]. The cultural connections between Australian Aboriginals and dogs [39,40] intensifies the wickedness of this issue. The ownership and management of dogs and cats involves multiple government, Indigenous, non-Indigenous and Traditional Owner stakeholders with potentially differing values and goals, and varied perspectives on how to address their growing numbers and associated potential health risks.

**Figure 1:**
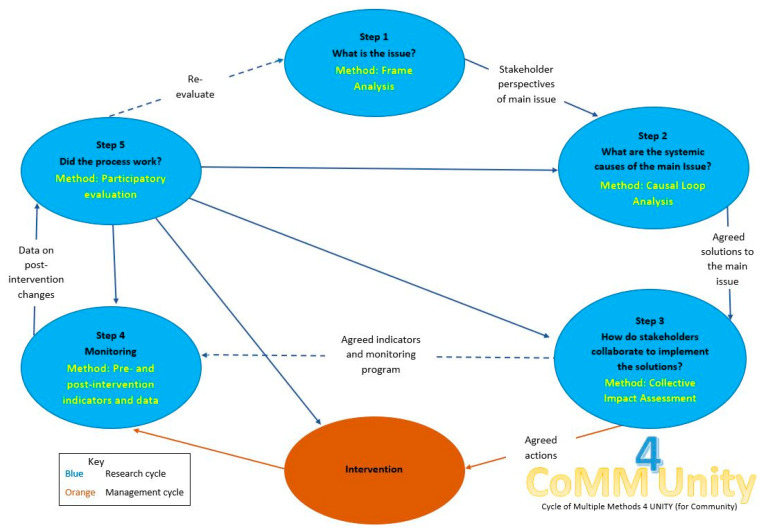
The CoMM4Unity approach, developed as a participatory action research process.

We have developed a participatory action research process, the CoMM4Unity (cycle of multiple methods for unity) approach (Figure 1), to provide a structure for stakeholders to utilise when attempting to solve a complex companion animal issue. In this case, we have adopted Burns’ [41] “systemic action research” concept, which responds to intractable problems which have proved resistant to simple solutions by engaging multiple stakeholder’s knowledge through participatory learning. This differs from more conventional action research in two important aspects. First, it purposefully engages with multiple actors across different institutional levels, rather than individuals alone. Second, it encourages group deliberation and learning about systems, defined by Burns [41] as “the interconnections between people, processes and the environment within which they are situated”. This includes encouraging understanding about system dynamics, feedbacks, vicious cycles and underlying “root” causes of problems, and the collective identification of solutions that can generate change. We have combined this with an adaptive decision-into-action cycle, which is commonly advocated to encourage communities to combine their knowledge to understand and address complex problems (e.g., [10,21]).

In this paper, we present a methodology and example of the first step in the CoMM4Unity process, which engages multiple stakeholders in the system and poses the question “What is the issue?”, in order to understand the stakeholder groups involved, their perspectives, knowledge and goals (Figure 1). First, stakeholders involved in animal management in the remote, island rAc of Wurrumiyanga, Tiwi Islands were identified, and then Gray’s [30] modification of frame analysis for environmental disputes was applied to analyse their perspectives and power dynamics to determine their relative positions on the problem. This was augmented by an assessment of the knowledge types held by stakeholders, and the power dynamics related to them.

## 2. Materials and Methods

### 2.1. Study Site

The Tiwi Islands are made up of 9 uninhabited islands and 2 inhabited islands located 60 km north of Darwin in the Northern Territory (NT) of Australia. The residents of Tiwi, whom are 90.8% Aboriginal and/or Torres Strait Islander, mostly live in one of the three main communities; Milikapiti (401 residents) and Pirlangimpi (371 residents) on Melville Island and Wurrumiyanga (1563 residents), the Islands’ capital, on Bathurst Island [42].

This research focuses on the community of Wurrumiyanga and their companion animals. Dogs, and more recently cats in rAcs, are valued companions with a unique style of ownership [29]. They can be “owned” not only by individuals but households and larger family groups, with the animal having free range to roam between all family households. Defining these as owned, free-roaming dogs and cats that are fed by humans but also having access to wildlife. Differing from feral dogs and cats that live outside the town borders with no or indirect (rubbish tip) contact with humans. Dogs are considered as family members who can choose their own actions, rather than owned pets [29,43]. There are also strong cultural connections to dogs in Tiwi culture, with dogs playing roles in ceremony and connections to ancestors through naming. With no Veterinary service on the Islands, it is difficult for the locals to efficiently take care of their animals. Veterinary services to the Tiwi islands are supported by AMRRIC (Animal Management in Rural and Remote Indigenous Communities), a not-for-profit charity established to facilitate companion animal health programs in rAcs in Australia [29,44]. These programs include surgical desexing, medication for parasites and humane euthanasia on request delivered alongside educational resources. Two of the authors (BK and WB) have participated in multiple dog health programs in Wurrumiyanga since 2013, as AMRRIC volunteers or as researchers.

Although no level of government in the NT formally holds the animal management portfolio, the Tiwi Islands Regional Council (TIRC) have contracted a one-week veterinary service for the Tiwi Islands bi-annually for the past two decades. The same AMRRIC-approved veterinarian has primarily been contracted for the last 22 years, over which time he has gained the trust of the Tiwi locals. However, other interventions have sometimes complicated the relationship between locals and animal management providers, such as the culling of dogs without the consent of the owner or the community. Recently, in 2018, by-laws for keeping dogs were introduced for the Tiwi Islands, requiring mandatory dog registration and the restriction of two dogs per household [45]. The TIRC has also introduced a USD 50 fee for community members to receive veterinary services (de-sexing and anti-parasitic medication) for up to two dogs, to help cover the cost of contracting the veterinarian [46]. The Tiwi Lands Council, and their Land and Sea Rangers, are responsible for the management of animals outside the borders of the communities including feral cats, dogs, pigs and horses. They do not have any management of companion animals inside the community boundaries.

### 2.2. Materials and Methods

Potential stakeholders in Tiwi Islands animal management were initially identified through discussions with Tiwi locals and Traditional Owners (TOs) that we had developed relationships with during previous visits as AMRRIC volunteers or researchers. Individuals were then approached and invited to participate in the project. Stakeholders fell into 10 initial groups; TOs, Wurrumiyanga community members, community youth, Indigenous Land Rangers, AMRRIC, private veterinarians, the NT Government’s Environmental Health Department, Tiwi Islands Regional Council (TIRC), NT Education Department, and the Red Cross (Table 3).

A total of 37 individuals were approached, but only 20 were available for interview, largely due to logistical constraints of contacting many people in the rAc, where telecommunication services were limited. Structured interviews were conducted using methods approved by CSIRO’s Social Science Human Research Ethics Committee (reference 137/17). Informed consent was given by all participants, however, one Tiwi community member did not wish to sign the consent form, therefore, the consent form was read out aloud and verbal consent was given and audio recorded. All interviews conducted with the 14 Wurrumiyanga residents took place face-to-face in a place chosen by the interviewee and where they felt comfortable. Prior and informed consent was requested and given by participants to audio record all interviews using a simple recording app, Voice Record Pro, on the interviewer’s iPhone 8. During each interview, questions (Table 4) were asked in the same order for each participant. Interviews took up to 60 min. Interviews were also conducted with the six non-residents of Wurrumiyanga. These were conducted online via Skype for Business and were audio recorded with their prior written consent. During these interviews, questions (Table 5) were asked in the same order for each participant, and also took up to 60 min.

### 2.3. Data Analysis

Audio recordings of the interviews were transcribed manually and uploaded to NVIVO. Discourse analysis [25] was utilised to examine each interview using a grounded theory approach, which allows themes and frames to emerge, rather than forcing them into preconceived categories [47]. Initially, a simple word count was conducted across all interviews to identify which subjects that the interviewees mentioned the most. Based on these results, grounded theory was then applied by coding for the themes emerging from the word counts. During the coding process, new themes also emerged that had not been expressed in the word counts.

Once all interviews were coded and new themes identified, coding with all themes was repeated for each interview. All coding was conducted by one author (BK), to mitigate differing coding interpretations. Using the Project Map tool in NVIVO, individual interviewees were mapped against all themes to compare results between interviews. Following this, the main themes were coded in more detail to further separate interviewee categories. For example, all sections coded “dogs” were coded into sub-themes for the type of discussion regarding dogs (e.g., positive, negative or neutral discussions), and all sections coded “dog issues” were then re-coded into specific issues (e.g., noise/barking, knocking over bins/rubbish, aggression towards people).

Having completed this analysis, the initial assignment of interviewees to the 10 stakeholder groups was re-visited, and a final grouping was made based on values and goals regarding animal management. Brown and Lambert’s [7] knowledge types (Table 1) and Gray’s [30] power frame and identity frame categories, whole-story frames and characterisation frames (Table 2) were attributed to each of the four newly-identified groups, again using the grounded theory approach.

## 3. Results

### 3.1. Participants

**Figure 2:**
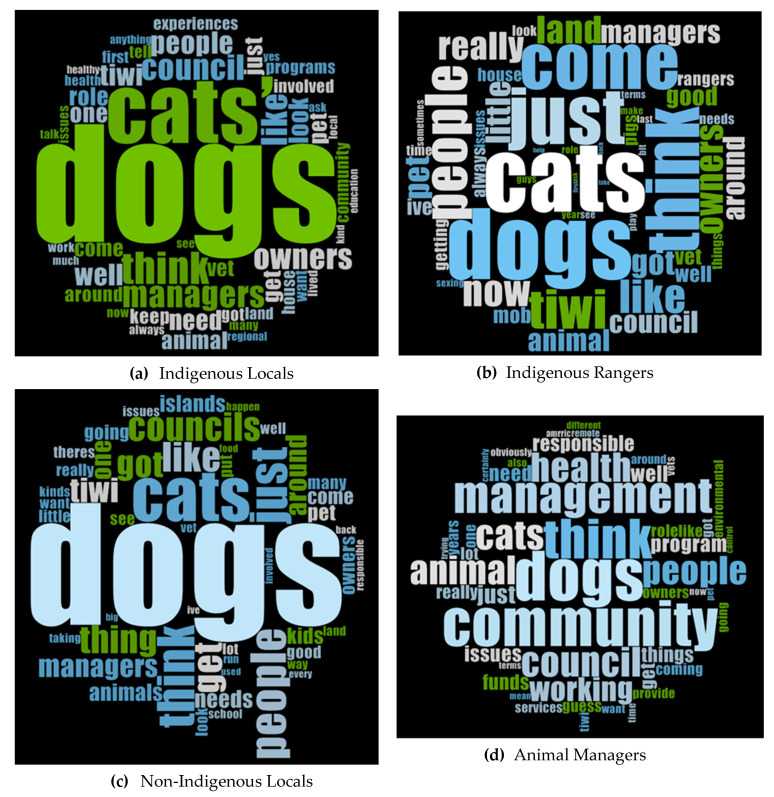
Word Clouds created in NVIVO showing the 50 most frequently occurring words from the interviews of the four final stakeholder groups: (**a**) Indigenous Locals, (**b**) Indigenous Rangers, (**c**) Non-Indigenous Locals and (**d**) Animal Managers.

The Project Map tool in NVIVO did not yield clear differences, as the themes generated were common amongst most interviewees. However, when separated further into sub-themes, it was clear that the values and goals behind the themes did vary amongst four groups: Indigenous Locals, Indigenous Land Rangers, Non-locals and Non-Indigenous Locals. Word counts were then conducted again, this time across the four new groups, and were transformed into Word Clouds in NVIVO for visual ease (Figure 2).

Based on these results, the original 10 stakeholder groups were refined into the four groups. Knowledge types, power frames and identity frames all aligned across these four groups. Some groups had individuals with multiple knowledge types and frame categories, however there were certain ones that were identified amongst all members of each of the four newly created groups.

### 3.2. Indigenous Locals

#### 3.2.1. Identity Frame

This group consisted of seven local Indigenous community members; TOs and community members, including two youths, and all resided in Wurrumiyanga and owned dogs and cats. All members had lived in Wurrumiyanga for most of their lives, except for one who grew up on his home country in Arnhem Land, NT, and moved to Wurrumiyanga more than 10 years ago but had visited many times throughout his life. The two youth participants did not have clear opinions about animal management in the community, whereas the remaining six had strong knowledge and views.

#### 3.2.2. Knowledge Type and Power Frame

All members of this group have individual and local knowledge. Individual knowledge has come from their own experiences growing up with and/or around animals in Wurrumiyanga. Their local knowledge comes from some of these same experiences that were shared together as a community, particularly around the issues currently impacting the community regarding animal management. All members of this group were participating in some aspect of animal management, they were all pet owners, and all agreed to participate in this study giving them the power of voice.

#### 3.2.3. Whole Frame

Dogs were discussed twice as often as cats by this group (Table 6, Figure 2a). Dogs were discussed freely, whereas cats were only discussed when asked about them, and when mentioned views about cats were more positive. The group did not think cats were an issue because they “never came across any cats” (Interviewee #6); “Yea you don’t see much cats here” (Interviewee #11); or “we don’t see many cats though; they must be at home” (Interviewee #14).

Conversely, dogs were discussed frequently. Positive discussions involved stories of pet dogs and why the local community own dogs, for example: “they were wonderful dogs, and they good hunters too” (Interviewee #12); “we keep them for protection and hunting and family members” (Interviewee #4); “people come and prowl around and yea dogs are protective too” (Interviewee #14); “they’re a part of the family and all we need is more management and education” (Interviewee #4).

The dog issue discussed the most was that there are ‘too many dogs’ (n = 11 references): “but dogs, they’ve just grown in numbers” (Interviewee #6); “there is too many dogs” (Interviewee #4). The group also knew of recent discussions about introducing dog by-laws, and everyone that mentioned this agreed that dog numbers needed to decrease, for example: “maybe reduce the dog numbers you know…just lower the numbers of the dogs within their house” (Interviewee #6); “too many, they only need one every house” (Interviewee #14).

“Cheeky dogs” (n = 9) and (poor) “health” (n = 6) were also common issues discussed. “(My son) is a dog lover…we went to the football oval…to have a catch of the ball and the dog just turned around and almost bit him on the face. And I turned around to the owner and said, ‘you should have kept that dog at home if it’s like that’” (Interviewee #11). “Some dogs you know got sores on their body, lots of dogs…the dogs need more treatment more medicine more often, keep them healthy” (Interviewee #14).

Although there are no Tiwi cultural lores that would hinder animal management programs for dogs and cats generally, this does not necessarily apply to all animals, as it was mentioned that “some dogs you can’t…because they’ve been named by their grandfather or father or aunty… because they’ve got a cultural name, so we’ve got to be careful. Only certain dogs” (Interviewee #12).

#### 3.2.4. Characterisation Frame

Indigenous Locals believed that the TIRC was doing a good job, because it brought a veterinarian to the island from Darwin to improve dog health. However, they felt that more needs to be done between veterinary visits, such as helping injured dogs and applying medications: “The ones that are needing help the council members should do something about it” (Interviewee #11), and “look after the animals and to give them medicine like that worm stuff…like when the vet isn’t here” (Interview #8). There was some debate about the role of the TLC: half of the group pointed out that the TLC only work outside the community and it is not their job to manage dogs, whilst the other half suggested that they should have a role in animal management similar to the TIRC. The group also concurred that the TOs have a role in educating the community about animal management.

#### 3.2.5. Summary

This stakeholder group’s overall framing was that dogs are a bigger problem than cats. The primary issue was that there are too many dogs, and that they should be managed in a culturally appropriate way by the TIRC and TLC working together, and TOs educating the community about what work is happening. This would reduce the number of dogs, reduce the number of cheeky dogs and improve the health of the remaining dogs.

### 3.3. Indigenous Rangers

#### 3.3.1. Identity Frame

The two local Indigenous Land Rangers that participated in the study had both lived in Wurrumiyanga for most of their lives and owned pets. Similar to Indigenous Locals, this group shared location and demographic characteristic identities, however, their position as Land Rangers offered a Role identity that was evident in their responses.

#### 3.3.2. Knowledge Type and Power Frame

The members of this group, like the Indigenous Locals, had individual and local knowledge based on their experiences growing up with animals in Wurrumiyanga. However, what separated the two participants was their specialised knowledge and their power of expertise. Their knowledge and power stemmed from their positions as Land Rangers on the Tiwi Islands. Land Rangers undertake natural resource management (e.g., weed control, feral animal control) on land outside the settlements and report to the TLC. This specialised knowledge of animals, their populations and how they interact with the environment, including the island settlements, gives them imperative knowledge and experience regarding animal management.

#### 3.3.3. Whole Frame

As for the other groups, Indigenous Rangers discussed dogs more frequently than cats (Table 6), and due to their role in the community, they discussed other species (e.g., feral pigs, buffalo and horses) more frequently than any other group.

Dog discussions were mostly positive rather than negative, whereas cats were regarded more negatively (Table 6). However, the negative references towards cats were about feral cats. Due to their roles as Rangers, they came across feral cats more often than the general community, possibly explaining this emphasis (Figure 2b).

The positive dog references were stories about owned dogs: “I can remember I had a little Corgi as my very first pet… I’ve got really good fond memory of that Corgi dog that we had” (Interviewee #9). The Indigenous Rangers also clarified why the locals have dogs, for hunting—“[my dogs] were good hunters anyway, pigs, possum, wallaby, sometimes they fight cats too” (Interviewee #13)—and for protection—“I like them because people come around at night driving around and the dogs let me know that somebody’s coming” (Interviewee #13).

The main negative discussions regarding dogs were about the health of the dogs, which the group believes should be the owners’ responsibility: “it falls back more or less on the owners of the animal you know make sure they’re aware of what it is and address it you know if they’ve got ticks address it” (Interviewee #9”), and “some people they don’t feed them, they go from the house to the bin, knock over bin and make rubbish everywhere” (Interviewee #13).

#### 3.3.4. Characterisation Frame

Both agreed that the TLC and the TIRC should work together to tackle animal management in Wurrumiyanga: “We both [TLC and TIRC] need to work out how to enforce new policies. The TOs also need to stand up and tell people to follow the new policies” (Interviewee #9). Involving the TOs will ensure local Indigenous knowledge is viewed and that the policies created will be culturally appropriate. They also thought that the TIRC was having a positive impact by bringing a vet from Darwin to treat dogs.

#### 3.3.5. Summary

In summary, Indigenous Rangers believed that animal management needs to occur across all species (dogs, cats, pigs, horses etc.). They believed that the TIRC is currently contributing to animal management by bringing a vet to the island from Darwin, but more needs to be done. This included more collaboration between stakeholders and ensuring that new policies are properly enforced to enable the animal management program to be more successful and sustainable.

### 3.4. Non-Indigenous Locals

#### 3.4.1. Identity Frame

Non-Indigenous Locals consisted of five non-Indigenous Wurrumiyanga residents. Two were pet owners and three were not; one had been in Wurrumiyanga his whole life and spoke the native language, whilst one had only been in Wurrumiyanga for 2 years.

#### 3.4.2. Knowledge Type and Power Frame

All members of this group had organisational knowledge and positional power. Although their knowledge originates from differing organisations, each have a role to play in animal management. Three work for TIRC who are responsible for delivering council policies. Another is a Councillor which gave him direct authority in developing policy. The Councillor also had individual and local knowledge from growing up in Wurrumiyanga. The fifth was a member of staff in the Department of Education NT and had organisational and specialised knowledge that could be used with positional power to educate school children about pet husbandry and personal health.

#### 3.4.3. Whole Frame

The group mentioned dogs more than twice as frequently as they mentioned cats (Table 6, Figure 2c). Negative discussion regarding dogs occurred more than positive discussions (Table 6). All five participants said that there were too many dogs in Wurrumiyanga, which result in other issues—roaming, human health risks, mess, aggression towards humans and dog fights. Statements included: “there’s too many dogs and not enough food… if there’s too many, they’re unhealthy… and they are always hungry” (Interviewee #7); “half the reason there’s rubbish on the street is because there’s dogs running around tipping over rubbish bins” (Interviewee #1); “they will tip over all the rubbish bins” (Interview #5), and “we’ve got underfed dogs fighting over a chicken bone out on the street” (Interviewee #1).

Relating to the fact that dogs are free-roaming, statements included: “they’re not dogs that are contained, nobody closes the gate, so they just wander around everywhere” (Interviewee #1). It was mentioned that dogs join to form packs, and then become more aggressive: “they roam everywhere they roam in packs if there is a small dog on its own it gets ripped to pieces” (Interviewee #3); “we’ve already got packs of dogs roaming around Wurrumiyanga, as soon as you’ve got packs running around, you’ve got too many dogs, there’s too many dogs, if you’ve got a pack of dogs you’ve got too many dogs. That’s why there’s a pack roaming around” (Interviewee #1); “I’ve been chased by one pack of dogs” (Interviewee #2) and “I rode a bike so of course I was chased by dogs” (Interviewee #1).

Dog health was also of concern, for example: “dogs are covered in ticks” (Interviewee #1), and “too many ticks on dogs and fleas on dogs… and if I was walking around with ticks all over me, I wouldn’t be very happy” (Interviewee #7). “Sometimes the cats and the dogs have sickness, they carry sickness” (Interviewee #7), which has an impact on human heath: “there’s dog poop everywhere they’ve got hookworms, roundworms, they’ve got every other worm, nobody worms these children” (Interviewee #5).

Cats were mainly seen in a negative light, with the major issue being their impact on native wildlife. This was followed by concern about there being too many cats, their potential to become feral, their high reproduction rate and potential zoonoses. For example, statements included: “cats (are) a massive issue because if they go feral and go into the bush and start destroying a unique environment that’s been protected by its isolation for so long, that has the potential to destroy so much of its natural beauty and characteristics” (Interviewee #2), “Tiwi islands have 53 indigenous bird species; we won’t have 53 indigenous bird species if we keep bringing cats onto the island. Cats eat birds it simple.” (Interviewee #1), “they go out and kill wildlife and they’re out at night… the damage they do, a lot of it is hidden and when its hidden people think you know because it’s not obvious that nothing needs to be done” (Interviewee #7). No positive discussions took place about cats.

#### 3.4.4. Characterisation Frame

Non-Indigenous Locals discussed that the TLC’s role does not include direct responsibility for community matters, but they do have a mandate to manage feral animals, including feral cats. It was clear that the group believed that the TLC should be responsible for cats because they are impacting on wildlife and biodiversity, while the TIRC should be managing dogs within the community. Vet programs on the island had been funded by the TIRC and provided surgical sterilisation (de-sexing) and parasite medications free-of-charge to the community. Three of the group felt strongly that the vet tax was not workable because none of the community’s dog owners can afford it, which is contributing to dog population growth. This group also strongly believed that TOs should be involved in dog and cat management, but currently they are not.

#### 3.4.5. Summary

The Non-Indigenous Locals’ framing can be summarised as follows: there are too many dogs in Wurrumiyanga, and this needs to be tackled by reducing the dog population, which will make management easier. Moreover, dogs can then be cared for better, with improved feeding, health care and hence reduced numbers of aggressive dogs and their risk to people. All stakeholders need to collaborate to achieve this, and the USD 50 vet tax is unworkable and should be terminated. This group also believe that cats are a major environmental problem that needs to be better managed, potentially by the TLC.

### 3.5. Animal Managers

#### 3.5.1. Identity Frame

The Animal Managers group consisted of seven non-residents of Wurrumiyanga, who all had current official positions related to animal management in Wurrumiyanga. This included two NT Government employees from the Environmental Health Department, three employed by AMRRIC and one private veterinarian based in Darwin who provided veterinary services to Wurrumiyanga.

#### 3.5.2. Knowledge Type and Power Frame

All members of this group had specialised knowledge and power of expertise. They had a range of roles relating to animal management in Wurrumiyanga. The veterinarian had been contracted by the TIRC to deliver veterinary services to Wurrumiyanga for the past 20 years. Two are NT Government staff under the Environmental Health Department, that supervise local staff located in remote communities.

#### 3.5.3. Whole Frame

This group discussed dogs and cats more equally (Table 6) and had a more holistic view of animal management, including multiple facets such as dogs, cats, community, management and health (Figure 2d). Some participants shared positive stories of dogs in remote communities and their cultural and social connections to the people (Table 6). For example: “dogs are treated as a responsible adult…someone who has their own right to exist, with responsibilities but can also make their decisions and are responsible for their actions… whereas in mainstream society dogs are treated more like a baby or child” (Interviewee #6).

However, negative opinions predominated (Table 6). For example: “there is just too many dogs” (Interviewee #20); “population numbers need to be attended to more so than they are” (Interviewee #17), especially because “with many dogs around dogs fight for resources” (Interviewee # 15). This results in dogs becoming aggressive: “dangerous dogs…put people at risk” (Interviewee #20), and “they are a concern around safety around my staff going out” (Interviewee #19). The dogs “try and attack cars and tear off mud flaps and even puncture tyres” (Interviewee #15); “dogs limit where you can walk” (Interviewee #18), and “(students) can’t go past a house with a cheeky dog to get to school, you have a restriction on lifestyle because of free-roaming dogs their pack formation so it can be dangerous to be jogging around or cycling around, especially on your own and it’s a big no-no at night time” (Interviewee #17). Most discussed the health issues of dogs and their roaming behaviours “they’re always free-roaming despite there being fences” (Interviewee #17) and “lesser numbers of dogs will reduce the amount of free roaming dogs” (Interviewee #16).

Cats were also discussed mainly in negative terms (Table 6). All six interviewees discussed the increasing numbers of cats across many remote communities including Wurrumiyanga, and they had strong opinions that cats will have a detrimental impact on local wildlife once feral. They believed this to be imminent because of the lack of local awareness about cats’ “high reproductive capacity and their survivability without human intervention (which) means that their potential to overflow from community is enormous” (Interviewee #15). Others mentioned that “I think (cats) are becoming over-populated drastically” (Interviewee #18), because “the (locals) are ignorant of the infinite capacity of cats to reproduce” (Interviewee #17). Cat-related zoonoses were also discussed, for example: “we don’t properly understand the burden that zoonotic disease is contributing, it might be significant or it might be negligible and until we know that burden we will advocate that we need to take a precautionary approach and that preventing zoonoses through basic worming and things that we can easily do is important for human health as well” (Interviewee #15). There were some positive discussions about cats in terms of companionship, and their usefulness for pest rodent control (Table 6).

A major point of discussion for this group was the responsibility for animal management in the islands. One stated: “it’s tricky in the NT at the moment in that there is no formal responsibility to include animal management… Regional Councils do acknowledge and accept that animal management is important for a community’s function and amenity and health” (Interviewee #15). The group believed that it should be the TIRC’s responsibility: “it can only really be the Regional Councils… it could only be who is on the ground and they need to get the adequate funding to do it” (Interviewee #20).

Many factors affected this group’s animal management work in communities. The group discussed three: the remoteness of the communities, the rapid turnover in government staff they work with, and the turbulent histories of many rAcs which impede the development of trusting relationships amongst animal management stakeholders.

#### 3.5.4. Characterisation Frame

It was discussed that the TIRC should “collaborate with other services such as Ranger groups” (Interviewee #18), and particularly the TOs, since they “are really key to it all” (Interviewee #20), “they are the voice for the community, leaders of the community” (Interviewee #18). Another stated: “it’s their land so absolutely they have a role and it’s important to work with them to make sure they’re happy with and to treat them with appropriate respect especially if they’re not happy with what’s going on it’s going to fail” (Interviewee #16).

#### 3.5.5. Summary

The Animal Managers’ framing can be summarised as follows: they believed that there are too many dogs, and an increasing number of cats. Cats must be controlled to avoid them becoming feral and impacting wildlife, and more needs to be known about their zoonotic impact on humans. Dog numbers should be reduced to manage their health, aggression, roaming behaviour and risks to human health. The statutory responsibilities for animal management also need to be clarified, plus sources of funding, and greater collaboration and coordination is required amongst all stakeholders, especially local community members, to create a sustainable animal management program.

## 4. Discussion

Differences among stakeholders’ values, goals and worldviews create conflicts which are the basis of intractable environmental problems [1,2,3]. Many of these can become “wicked problems”, whereby no true definition, outcomes or resolution can be agreed upon, causing further conflict. Stakeholder participation is necessary to solve such complex problems, because multiple knowledge cultures are needed to understand and intervene. Furthermore, knowledge cultures are also influenced by power, which determines collective learning processes and their ultimate outcomes [5,7,28].

However, methods are still required to better characterise stakeholders’ different perspectives and power dynamics, which are essential if effective processes are to be designed, and solutions found to environmental conflicts. This is particularly necessary in Australia, where the knowledge and values of Indigenous people have been devalued relative to the scientific perspectives of researchers and politicians [48]. Some innovative research has begun to tackle intractable environmental disputes in partnership with Indigenous peoples, in order to apply their knowledge, values and perspectives, and to integrate these respectfully, equitably and constructively with other knowledge cultures [14,17,18,19,20]. However, specific methods to map power differentials during collective learning processes remain weak [16,49].

To address this gap, we applied Gray’s [30] frame analysis, combined with Brown and Lambert’s [7] concept and typology of knowledge cultures to characterise stakeholders’ perspectives of animal management in the rAc of Wurrumiyanga. This forms the first step of our participatory systemic action research process, the CoMM4Unity approach, and aims to understand the stakeholder groups involved, their perspectives and goals around the shared challenge of improving companion animal management. Due to the remoteness of the study area, limited communication infrastructure, and the dispersed nature of the stakeholders involved, only 20 interviewees were engaged, yielding four groups. While this undoubtedly limited the depth of our analysis, there were still sufficient data to differentiate between the frames of the groups. Hence, our results suggest that the method has the potential to be used in any conflict situation where multiple stakeholders are involved, for example, seals and salmon in Scotland [50]. It may also assist in solving situations where cross-cultural stakeholder groups are involved, and cultural differences may be a barrier [2].

The results revealed four stakeholder groups with distinct identity frames, knowledge types and power frames. However, all four groups shared overlapping perceptions regarding companion animals in Wurrumiyanga, although with differing strengths of opinion about how they should be managed. Therefore, the situation is not one of diametrically opposing frames, as is often the case in natural resource management [2,50], but more a misalignment of goals and values. This may be linked to fundamental differences in beliefs, which underlie values [3], exemplified in this case by the differing understandings of the role of dogs and cats in Wurrumiyanga. For example, Indigenous residents of Wurrumiyanga (Indigenous Locals and Indigenous Rangers) spoke almost equally positively and negatively about dogs, and discussed their needs for the dogs, including as companions, for hunting and house protection, plus the problems caused by large numbers of dogs. By comparison, the non-Indigenous participants (Non-Indigenous Locals and Animal Managers) discussed dogs positively less than half as often as they did negatively, focusing on the problems caused by dogs.

This pattern was similar for cats, where Indigenous residents of Wurrumiyanga spoke of cats more positively than negatively, citing their benefits for rodent control and as companions. By contrast, the non-Indigenous participants spoke of cats in an extremely negative light, with 10-fold more negative than positive comments, focusing on their high fecundity and impacts on wildlife.

The frame analysis demonstrated that all interviewees agreed that the main overarching issue was dog overpopulation, but that the strength of their views differed. Unfortunately, this was not quantified, but during interviews, it appeared that the Indigenous Locals only answered the questions because they had been asked, not because they felt that the problem was so acute that they should become actively involved. The varied severity of suggested management actions also revealed the strength of different groups’ views. Indigenous Locals agreed that there were some problems with dogs, and that there are too many, but they only suggested reducing the population rather than introducing movement restraints, because they wished dogs to be able to accompany them to the shops or walk their children to school. Moreover, this group highlighted that dogs have cultural value and names that can’t be regulated with Western by-laws, and hence these animals cannot be interfered with. Their moderate and reluctant views may also have been influenced by past experiences, where researchers had engaged with them for personal or professional gain, and outside authorities had created animal management policies that the locals opposed [51].

By comparison, the Non-Indigenous Locals group had more forthright opinions, suggesting that animal management should be urgently improved, and that this requires not only reducing dog (and cat) numbers, but the enforcement of by-laws similar to Western societies, including pet registration, constraints on pet roaming and their improved health care. This group wanted pet owners to take more responsibility for their companion animals. These values differed from those of Indigenous Locals, who regarded dogs as adults who have the same rights and responsibilities as people, and therefore dog control is tantamount to the removal of their rights. The majority of the Non-Indigenous Locals group had positional power in Wurrumiyanga, whereby their occupation (TIRC) or elected role (Councillor) gave them authority to create and enforce policies, including those regarding animal management, and they have been involved in successful animal management decisions in the past [52]. However, these successes were short-lived since gains were quickly lost after the researchers had departed, perhaps because an inclusive participatory research process had not been carried out. Another reason for this may be that as a community in the NT, no level of government is responsible for animal management, and therefore resources are used on issues that are labelled mandatory, leaving little or no resources for animal management.

With their role as natural resource managers on Tiwi, the Indigenous Rangers had a more holistic view and knowledge of animals and their management, and therefore, their framing fell somewhere between the Indigenous and non-Indigenous local groups. The Rangers wanted to see an improvement in dog and cat management but wanted any solutions to be appropriate for the community. Some researchers claim that Aboriginal community values and knowledge shouldn’t be as respected as scientific or Western values, because they lack the overall knowledge and understanding of the whole issue [53]. However, although this group clearly acknowledged the impacts of cats on wildlife [28] and their knowledge passed down from previous Rangers about the land, flora and fauna, they also believed that local Indigenous knowledge and values must play a key role in management.

The Animal Manager’s frame was similar to the Indigenous Rangers’ frame, being intermediary. This group had strong opinions that animal management needs to be improved but understood that for a program to be successful and sustainable, the community must be involved, and their cultural values need to be respected. They accepted that local knowledge is vital. This group had organisational knowledge regarding improving animal management in rAcs, as well as specialised knowledge regarding education and teaching, and as veterinarians. Their positions in these organisations and their expertise in their fields gave them positional and power of expertise frames. These knowledge types and power frames could be used together to make changes across multiple rAcs, and not only in Wurrumiyanga.

**Figure 3:**
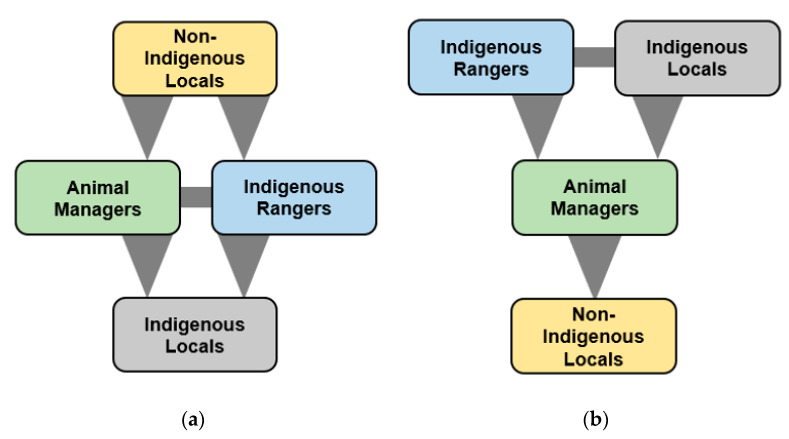
The relative ascendancy of the four stakeholder groups in terms of (**a**) Power Frame and (**b**) Knowledge Type. Groups are placed from high to low in terms of power and influence, and arrows reflect the direction of the relationships.

In terms of the relative power of the four groups, Non-Indigenous Locals held the ascendancy over the other three groups due to their Positional Power Frame, which gave them direct authority to create and enforce policy relating to animal management (Figure 3a). Animal Managers and Indigenous Rangers were secondary and had equal levels of power with expertise in the animal field, having greater influence than Indigenous Locals through their power of voice. By comparison, in terms of Knowledge Types (Figure 3b), the roles were reversed, with Indigenous Rangers and Indigenous Locals having the greatest but similar knowledge, Individual and Local, as a result of growing up in Wurrumiyanga, living with these animals on a daily basis and having had cultural traditions passed down to them through generations. This superseded the specialised knowledge type of Animal Managers that covered a broad range of animal management skills, but lacked Local knowledge specific to Wurrumiyanga and the cultural lore under which the locals are governed. The organisational knowledge of the Non-Indigenous Locals is subsumed by other stakeholder groups, as this relates more to how to create policies, rather than what policies to create. Thus, when taken together, the net effect was that all four groups had similar levels of power, but for different reasons. This also reflects the overall conclusion of the frame analysis, that there are no polarized views and major power asymmetries regarding animal management within Wurrumiyanga, but rather a diversity of similar views, power frames and knowledge types relating to the problem.

## 5. Conclusions

Frame analysis has not been used before to diagnose the underlying power dynamics in natural resource or environmental conflicts in Indigenous Australia. Our application showed that it can reveal subtle variations in stakeholder groups’ identities, knowledge, power, goals and values. The method was relatively resource efficient and tractable despite the remoteness of the study site, and the wide geographical distribution of the stakeholders initially identified. This influenced the sample size of interviewees, and therefore the depth of analysis that was possible, but this is likely to be typical of remote regions such as the NT where stakeholders are dispersed over large distances and with limited communication infrastructure. By integrating Brown and Lambert’s [7] concept of knowledge types, our frame analysis also highlights the kinds of knowledge that these different groups can bring to a collective learning process, and potential solutions to the complex challenges of companion animal management in the study site. Interestingly, in this case, the frame analysis revealed that the problem is not as conflicted as perhaps first assumed. Rather, the four stakeholder groups maintained overlapping views of the same problem, but with differing strengths of opinions about potential improvements to management and the institutions and partners who should be involved. This method has the potential to be used in any conflict situation where multiple stakeholders are involved, including those where cross-cultural stakeholders may be a barrier. This lays the foundations for the following steps of any participatory action research process, such as our CoMM4Unity approach.

## Figures and Tables

**Table 1:** Different knowledge types, where knowledge is learnt, and the context in which they may be found. Modified from Brown and Lambert [10].

Knowledge Type	Where Knowledge Is Learnt	Where Knowledge Is Found	Sources of Truth	Sources of Ignorance
Individual	owned lived experience, lifestyle choices, learning style, identity	reflections learning	memory learning style five senses	subjective limited vague
Local	shared lived experience of individuals, families, businesses, communities	stories events histories	stories events symbols	gossip anecdote inaccurate
Specialised	mono, multi- and trans-disciplinary, the professions	case studies experiments	inquiry measurements observations	jargon irrelevant narrow
Organisational	governance, policy, and strategies of organisations	agendas alliances plans	agendas alliances networks	deals mates corruption
Holistic	core of the matter, a common purpose	symbol vision ideal	synthesis focus creative leap	airy-fairy impossible impractical

**Table 2:** Definitions of Power Frames and their categories, Identity Frames, Whole-story Frames and Characterisation Frames, modified from Gray [25].

Power Frame Categories	Definition
Voice	Power comes from participation, therefore having a voice at the table
Moral	Belief that one is ethically and/or morally “right”, giving themselves the power
Force	Power comes from forcing/threatening coercive actions
Sympathy	Victims (children, endangered species), are likely to be supported on an emotional level
Coalitional/relational	Power comes from membership of a group that supports a particular perspective
Personal	Power comes from one’s own interpersonal style (e.g., charisma, advanced computer skills, negotiation experience)
Expertise	Power from possessing relevant knowledge and experiences that other lack
Resources	Power from having resources (e.g., time, money, staff)
Authority/positional	Power from the actual ability to make decisions based on one’s role, job title, organisational position
**Identity Frame Categories**	
Demographics	Race, gender, ethnicity
Location	Where they live
Role	Occupation
Institutions	Local Government
Interest	All oppose the same thing
**Whole-story Frame**	A summary of what each party believes the dispute to be about, guiding the behaviour of each party
**Categorisation Frame**	Who are the other parties? One party’s expectations and evaluations of another party’s behaviour and/or attitudes, arising from the attributions of blame and causality

**Table 3:** The initial stakeholder groups and characteristics of the 20 interviewees, clustered into their refined groups according to their corresponding knowledge types and power frames.

Interviewee	Stakeholder Group	Location	Gender	**Age**
**Indigenous Locals**				
4	Traditional Owner (TO)	Wurrumiyanga	F	25–50
6	TO	Wurrumiyanga	M	>50
8	Community Youth	Wurrumiyanga	M	<25
10	Community member	Wurrumiyanga	M	>50
11	Community Youth	Wurrumiyanga	F	<25
12	Community member	Wurrumiyanga	M	25–50
14	Community member	Wurrumiyanga	M	>50
**Indigenous Rangers**				
9	Land Ranger	Wurrumiyanga	M	25–50
13	Land Ranger	Wurrumiyanga	M	25–50
**Non-Indigenous Locals**				
1	TIRC	Wurrumiyanga	M	>50
2	TIRC	Wurrumiyanga	M	25–50
3	TIRC	Wurrumiyanga	M	>50
5	NT Education Department	Wurrumiyanga	F	25–50
7	Red Cross	Wurrumiyanga	M	>50
**Animal Managers**				
15	AMRRIC	Darwin	F	25–50
16	Veterinarian	Darwin	M	25–50
17	AMRRIC	Darwin	F	>50
18	AMRRIC	Darwin	F	25–50
19	NT Environmental Health Department	Darwin	M	25–50
20	NT Environmental Health Department	Darwin	F	25–50

**Table 4:** Interview questions for individual residents in Wurrumiyanga, regarding animal management in Wurrumiyanga.

Questions
1. What is your background?
2. Can you tell me about your first experience with dogs and cats?
3. Can you tell me about your current experience with dogs and cats?
4. Who is involved in cat and dog management here on Tiwi?
5. Who should be involved in cat and dog management?
6. What is your relationship with (stakeholder), and do you think they have a role in animal management? (a) Tiwi Islands Shire Council (b) Tiwi Land Council (c) Traditional Owners (d) Pet Owners (e) Non-pet owners
7. What do you think that the managers are doing well?
8. What should the managers be doing differently?
9. What are the kinds of issues or challenges that you face in managing cats and dogs in rAcs?
10. Is there any cultural lore that you know of that would prevent certain cat or dog management programs from occurring?
11. If you were in my shoes, what would you ask; what would you want to find out more about?

**Table 5:** Interview questions for individuals that were non-resident in Wurrumiyanga, regarding animal management in Wurrumiyanga.

Questions
1. What is your background?
2. How long have you worked for…………….?
3. What is your position and responsibility?
4. What are the limits to your position?
5. Where does your funding come from?
6. What is your funding allowance?
7. What are your thoughts on dogs in remote Aboriginal communities (rAcs)?
8. What are your thoughts on cats in rAcs?
9. What is your role in dog and cat management in rAcs?
10. What is your experience with dog and cat management, and can you provide examples?
11. What are the kinds of issues or challenges that you face in managing cats and dogs in rAcs?
12. Who is responsible for cat and dog management?
13. Who should or should not be responsible for cat and dog management?
14. Why should dog and cats be managed?
15. How should cats and dogs be managed?
16. What is your relationship with the Wurrumiyanga Community?
17. What is your relationship with (stakeholder), and do you think they have a role? (a) Tiwi Islands Shire Council (b) Tiwi Land Council (c) Traditional Owners (d) Pet Owners (e) Non-pet owners
18. If you were in my shoes, what would you ask; what would you want to find out more about?

**Table 6:** The number (n) of times that a theme was discussed and coded in each stakeholder group’s interviews. Any dialogue relating to dogs and cats was themed as “general”, and then separated into sub-themes—positive and negative dialogue—with the remaining being neutral.

Stakeholder Group		Dog			Cat	
	General n	Positive n (%)	Negative n (%)	General n	Positive n (%)	Negative n (%)
Indigenous Locals	30	13 (43%)	17 (57%)	28	8 (29%)	4 (14%)
Indigenous Rangers	6	4 (67%)	2 (33%)	5	1 (20%)	3 (60%)
Non-Indigenous Locals	32	10 (31%)	22 (69%)	32	1 (3%)	24 (75%)
Animal Managers	20	5 (25%)	15 (75%)	25	3 (12%)	22 (88%)
